# Electrochemical Properties and Structure Evolution of Starch-Based Carbon Nanomaterials as Li-Ion Anodes with Regard to Thermal Treatment

**DOI:** 10.3390/polym11091527

**Published:** 2019-09-19

**Authors:** Marcelina Kubicka, Monika Bakierska, Krystian Chudzik, Małgorzata Rutkowska, Joanna Pacek, Marcin Molenda

**Affiliations:** Faculty of Chemistry, Jagiellonian University, Gronostajowa 2, 30-387 Krakow, Poland; lis@chemia.uj.edu.pl (M.K.); krystian.chudzik@doctoral.uj.edu.pl (K.C.); malgorzata.rutkowska@uj.edu.pl (M.R.); j.swider@doctoral.uj.edu.pl (J.P.)

**Keywords:** Li-ion battery, anode, carbon aerogel, starch, pyrolysis, electrochemical performance, structure evolution

## Abstract

The influence of the pyrolysis temperature on the structural, textural, and electrochemical properties of carbon aerogels obtained from potato, maize, and rice starches was analyzed. The carbonization of organic precursors, followed by gelatinization, exchange of solvent, and drying process, was carried out in an argon atmosphere at temperatures ranging from 600 °C to 1600 °C. The nanostructured carbons were characterized by X-ray powder diffraction (XRD) as well as N_2_-adsorption/desorption (N_2_-BET) methods. The electrochemical behavior of Li-ion cells based on the fabricated carbon anodes was investigated using the galvanostatic charge/discharge tests (GCDT) and electrochemical impedance spectroscopy (EIS). The results show that the thermal treatment stage has a crucial impact on the proper formation of the aerogel material’s porous structures and also on their working parameters as anode materials. The highest relative development of the external surface was obtained for the samples pyrolysed at 700 °C, which exhibited the best electrochemical characteristics (the highest specific capacities as well as the lowest charge transfer resistances).

## 1. Introduction

Although many different anode materials have been widely studied recently [1,2,3,4,5], carbonaceous materials are still the most commonly used in commercial lithium-ion batteries (LIBs) [6,7]. Their application as Li-ion anodes in 2016 was 96% of all anode types [8]. This is due to their favorable characteristics, such as desirable electrochemical potential, relatively high specific capacity, and excellent stability during cycle performance, as well as cost competitiveness and benign impact on the environment [9,10,11]. Nevertheless, to deal with the current energy challenge, research efforts should be devoted to utilizing various carbon materials based on sustainable resources that reveal an ability to obtain high-performance electrode materials [10,12,13,14,15]. In fact, naturally abundant precursors are not only affordable and inexhaustible but often possess hierarchical structure and specific porous organization and are thereby of high interest to derive advanced carbons that exhibit unique properties [14,16,17]. Currently, a large amount of investigations focus on the heat treatment of biomasses or industrial waste [10,13,14,16,17,18,19,20,21,22,23]. Our latest studies [12,24,25,26,27] deal with the development of the hierarchically arranged, nanostructured carbon aerogels (CAGs), derived from starch, the major carbohydrate reserve in plants, which is also one of the most abundant natural polymers on earth. This renewable and biodegradable polysaccharide generally consists of linear amylose and highly branched amylopectin [28]. The content and ratio of these two aforementioned units in starch vary depending on the botanical source and affect the properties of the end product, providing tailor-made materials [12,24,29]. Still, not only raw materials influence the characteristics of the final compounds, but also various preparation conditions, for example, different carbonization temperatures. However, most of the precursor materials are usually subjected to a pyrolysis in a relatively narrow temperature range or just at a single temperature, so almost no comprehensive research has been carried out relating to the material changes in terms of physico- and electrochemical features along with the thermal treatment [16].

In this work we report on in-depth and broad examination on the preparation and characterization of carbon nanostructures derived from starch precursors through a systemic heat treatment process from 600–1600 °C. A matter of particular concern is the correlation between the carbonization temperature and the evolution of the structure and electrochemical properties of carbon-based anodes that are required in order to achieve the best performance with the lowest energy consumption and low carbon footprint.

## 2. Materials and Methods

The CAG materials were synthesized via the gelatinization process of different types of starch (rice, maize, and potato, Sigma Aldrich, Saint Louis, MO, USA) followed by the solvent exchange, the drying process, and the carbonization of the obtained samples under argon (Air Products, 99.999%, Allentown, PA, USA) flow (described in detail previously by our group [12,24,25,26]). The temperature of the pyrolysis process was established at 600 °C, 700 °C, 900 °C, 1200 °C, and 1600 °C, with a constant heating rate (2°/min) and the temperature being maintaining for 6 h.

The phase purity of the synthesized materials was investigated by X-ray powder diffraction (XRD) using a Bruker D2 PHASER diffractometer (Billerica, MA, USA) with a Cu lamp, *K*_α1_ radiation, *λ* = 0.154184 nm, between 10° and 60° 2*θ* range with a step of 0.02°. Textural properties of the samples were characterized by N_2_-sorption at −196 °C using a 3Flex v1.00 (Micromeritics, Norcross, GA, USA) automated gas adsorption system. Prior to the analysis, the samples were degassed under vacuum at 350 °C for 24 h. The specific surface area (*S*_BET_) of the CAG materials was determined using the Braunauer–Emmett–Teller (BET) model. The external surface area and the micropores volume were calculated from the t-plot model using the Carbon Black STSA thickness curve.

The electrochemical behavior of CAG based materials pyrolysed at different temperatures was examined by the galvanostatic charge-discharge tests (GCDT) performed using the ATLAS 1361 MPG&T multichannel battery tester (ATLAS–SOLLICH, Rębiechowo, Poland) in a 0.001–3.0 V potential range, under different current loads. All cells contained metallic lithium (Sigma Aldrich, 99.9%, Saint Louis, MO, USA) as a reference, a trilaminate of polypropylene/polyethylene/polypropylene film (Celgard 2325) (Celgard LLC, Charlotte, North Carolina, USA), and two porous glass microfiber filters (Whatman GF/F) (Sigma Aldrich, Saint Louis, MO, USA) as separators and a solution of 1M lithium hexafluorophosphate (LiPF_6_) dissolved in a mixture of ethylene carbonate and diethyl carbonate (50/50 *v*/*v*, Sigma-Aldrich, battery grade, Saint Louis, MO, USA) as an electrolyte. The carbon electrodes were prepared by mixing 90 wt% of active material with 10 wt% of polyvinylidene fluoride (Sigma Aldrich, Saint Louis, MO, USA), used as binder, in N-methyl-2-pyrrolidone solvent (Sigma Aldrich, ≤99.5%, Saint Louis, MO, USA). The slurry was spread onto copper foil and dried in an air oven at 100 °C for 48 h under ambient pressure. The cells were assembled in an argon-filled glove box (MBraun glove box, Garching, Germany) with a high-purity atmosphere (H_2_O and O_2_ <0.1 ppm). Additionally, the electrochemical impedance spectroscopy (EIS) measurements were carried out on the potentiostat/galvanostat (AUTOLAB PGSTAT302N/FRA2, Metrohm Autolab, Utrecht, The Netherlands) at 3.0 V potential of the cells by applying 0.1 V amplitude, in a frequency range from 100 kHz to 0.8 Hz. The impedance data were fitted using Nova 1.11 software. 

## 3. Results and Discussion

**Figure 1:**
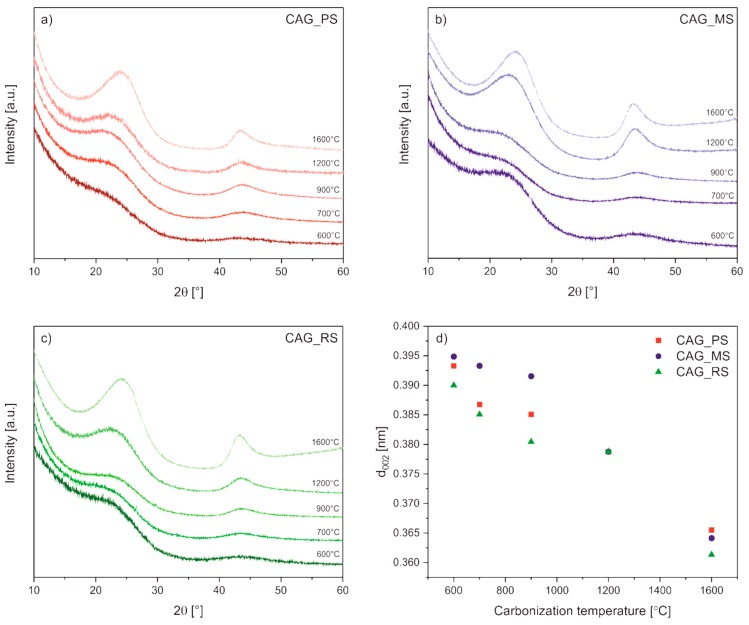
X-ray diffraction patterns of carbon aerogels derived from (**a**) potato starch (PS), (**b**) maize starch (MS), and (**c**) rice starch (RS) carbonized at temperatures ranging from 600–1600 °C as well as (**d**) the correlation between interplanar spacing *d*_002_ and the carbonization temperature of all carbon aerogel (CAG) samples.

Figure 1 shows X-ray diffraction patterns of carbon aerogel samples obtained from various types of starch (potato (PS), Figure 1a; maize (MS), Figure 1b; rice (RS), Figure 1c), pyrolysed at different temperatures within the range of 600 to 1600 °C. The XRD patterns exhibit two broad peaks at about 24° and 43° 2*θ*, which are assigned to the (002) and (100) crystal planes in the carbon structure, pointing out that the obtained CAG materials are composed of the amorphous phase with some contributions of graphitic domains [22,30]. As it can be noticed, with increasing carbonization temperature from 600 to 1600 °C, the (002) peak becomes sharper and gradually shifts right for all samples, which indicates not only a rise in long-range order degree but also a decrease in interplanar spacing *d*_002_ (Figure 1d), both relating to an improved degree of CAG graphitization induced by high temperatures. The d-spacing values decline slowly from 0.3933, 0.3949, and 0.3900 to 0.3655, 0.3641, and 0.3613 nm for CAG_PS, CAG_MS, and CAG_RS samples, respectively, but are still larger than that of graphite (*d*_002_ = 0.3355 nm) [31].

The textural parameters of the studied samples (presented in Table 1) were determined based on the nitrogen sorption measurements. In all cases of the series, specific surface area, external surface area, volume of micropores, and pore volume are growing with an increase of the samples’ carbonization temperature and are reaching maximum values at about 900 °C. Above this temperature, the mentioned variables start to decrease until practical destruction of the material structures at 1600 °C. The observed drop of the textural parameters of the investigated aerogels at high temperatures can be connected with the collapse of the samples’ pore structure and the sintering effect of the sample grains, which reduces the accessibility of the N_2_ molecules during the adsorption process. It is worth noting that, despite that, the highest BET surface areas were obtained from the carbon materials heated at 900 °C and the highest relative development of the external surface was achieved from those pyrolysed at 700 °C. This is well visible in the *S*_EXT_/*S*_BET_ ratio, which reaches the highest value for the starch based materials treated at 700 °C. The highest *S*_EXT_/*S*_BET_ ratio is accompanied by low *V*_MIC_/*V*_TOT_ values, which confirms the highest relative development of the external samples surface.

**Figure 2:**
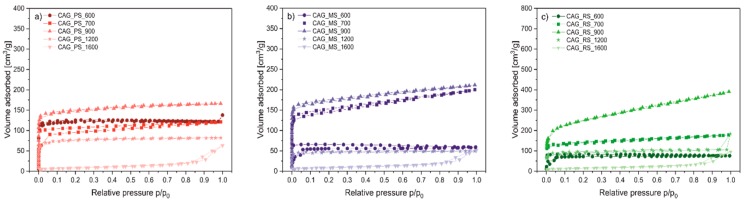
Nitrogen adsorption-desorption isotherms for the samples from (**a**) CAG_PS, (**b**) CAG_MS, and (**c**) CAG_RS series.

Nitrogen adsorption-desorption isotherms of the carbon aerogels from the PS, MS, and RS series are presented in Figure 2a–c, respectively. Excluding the samples calcined at 1600 °C, the isotherms of type I were obtained (according to International Union of Pure and Applied Chemistry classification) with the high increase of adsorbed nitrogen volume at low nitrogen partial pressures (characteristic of microporous materials). In the case of the samples calcined at 1600 °C, only adsorption at high nitrogen partial pressures was observed, which can be connected with interparticle porosity.

**Figure 3:**
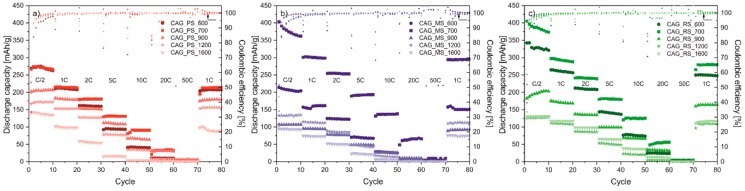
The rate capability of the (**a**) CAG_PS, (**b**) CAG_MS, and (**c**) CAG_RS based anodes at various C rates.

Galvanostatic charge-discharge cycling was performed to investigate the effect of thermal treatment on the electrochemical efficiency of carbon aerogels in a Li-ion battery. In Figure 3, the performance of Li/Li^+^/CAG cells is compared at different current rates from C/2, 1C, 2C, 5C, 10C, 20C, 50C, and 1C in 8 sets of 10 cycles at room temperature. The typical mass loading of active material in electrodes was established at about 1.42 mg/cm^2^, 1.46 mg/cm^2^, and 1.54 mg/cm^2^ for CAG_PS, CAG_MS, and CAG_RS based electrodes, respectively. For all analyzed cells, a specific capacity fade with increasing C-rates is observed, as expected. However, when the C-rate is returned back to 1C after 70 cycles of working, the capacity resumed back to initial values from the 10th cycle, which indicates a very good cycling stability of the studied carbon electrodes. What is more, after first capacity loss (ascribed to solid electrolyte interphase layer formation), a very good coulombic efficiency is achieved that illustrates the proper reversibility of Li-ion insertion/extraction reactions. As shown in Figure 3a–c, the highest values of specific capacities (even at high C-rates such as 10C or 20C) are reached by cells based on samples prepared at 700 °C, and the lowest values are reached by cells based on CAG_1200/CAG_1600, which is consistent with the BET results that identify structural collapse in these materials resulting in poor electrochemical performance. Generally speaking, appropriate surface development is highly desirable for the effective and stable operation of the cell. After the 80th cycle of working, the reversible capacity is equal to 214, 295, and 280 mAh/g consecutively for Li/Li^+^/CAG_PS_700, Li/Li^+^/CAG_MS_700, and Li/Li^+^/CAG_RS_700 as well as 89, 75, and 117 mAh/g for Li/Li^+^/CAG_PS_1600, Li/Li^+^/CAG_MS_1600, and Li/Li^+^/CAG_RS_1600, respectively, with the reversibility fluctuating between 99.9–100.4% in both cases. It follows that the pyrolysis temperature has a significant influence on the capacity, but it is not relevant for cycling stability. On the other hand, it is important to notice that although the CAG_MS_700 and CAG_RS_700 electrodes exhibit much better electrochemical performance in comparison to the CAG_PS_700 system, the deterioration in the specific capacity between the current sets is more emphasized for both of them. Notwithstanding, especially for the high current loads (such as 5C and 10C), carbon aerogels pyrolized at 700 °C achieve much higher capacities than commonly used graphite (regardless of starch origin) [27].

**Figure 4:**
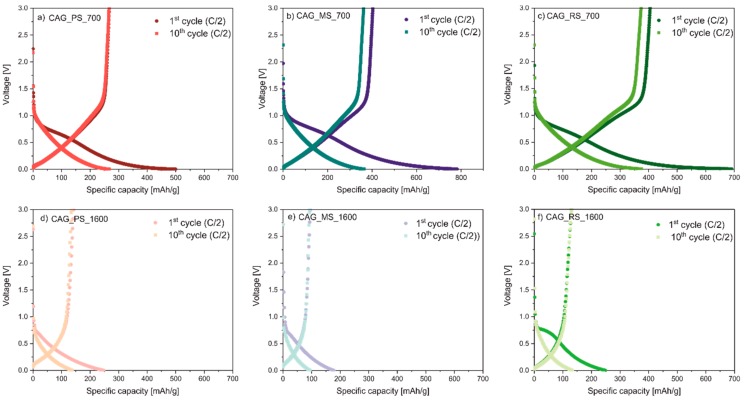
Charge-discharge voltage profiles for the first and tenth cycles of (**a**) CAG_PS_700 (**b**) CAG_MS_700, and (**c**) CAG_RS_700 vs. (**d**) CAG_PS_1600, (**e**) CAG_MS_1600, and (**f**) CAG_RS_1600 at C/2 current rate.

Figure 4 compares the shape of the voltage curves for the Li/Li^+^/CAG_700 and Li/Li^+^/CAG_1600 cells for the 1st and 10th cycles at C/2 current rate. The discharge/charge characteristics differ from the voltage profiles of typical graphite anodes. The potential curves show a discernible polarization between charge and discharge line (hysteresis), and high irreversible capacity is also observed. Thus, these materials can be ascribed to disordered carbons with some graphene domains, which was also confirmed by X-ray powder diffraction [32,33].

**Figure 5:**
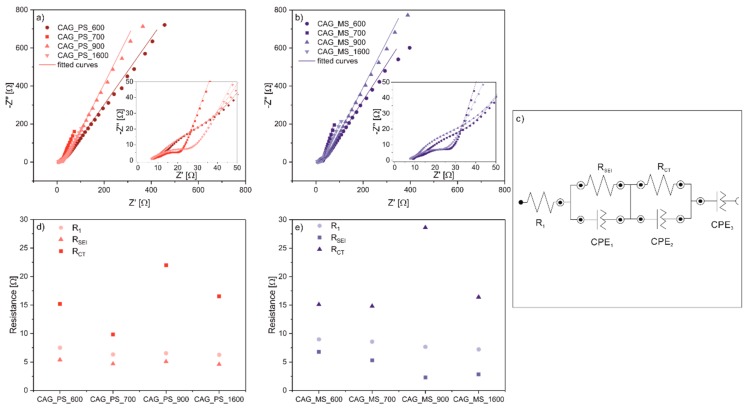
Nyquist plots for (**a**) Li/Li^+^/CAG_PS and (**b**) Li/Li^+^/CAG_MS cells with (**c**) the modeled equivalent circuits as well as the correlation between the R_1_, R_SEI_, and R_CT_ resistances values of (**d**) CAG_PS and (**e**) CAG_RS anode materials in Li-ion cell and their pyrolysis temperature.

In order to analyze the electronic and ionic transport properties of selected CAGs, EIS measurements were carried out after the 80th cycle of the discharging and charging tests. For the impedance tests, CAG_MS and CAG_PS based materials were chosen as the best and the worst performing representatives among the studied bio-derived aerogels. Nyquist plots (Figure 5a,b), presented for all measured cells, consist of three parts; one depressed semicircle at the high frequency range, a second depressed semicircle at the mid-to-low frequency range, and a straight line at the low frequency range. These impedance spectra can be simulated and interpreted through the following equivalent circuit (Figure 5c): *R*_1_- attributed to the uncompensated ohmic resistance of the cell, *R*_SEI_/CPE_1_-, connected with the first depressed semicircle and corresponding to the solid electrolyte interphase (SEI) properties, *R*_CT_/CPE_2_-, related to the second semicircle ascribed to charge transfer reactions and the last CPE_3_, assigned to the diffusion process. The values of *R*_1_ for all samples decrease with the growth of the carbonization temperature, nonetheless this change is negligible. As can be seen for both PS and MS carbon aerogels, calcination at 700 °C yields the lowest overall impedance of the system (Figure 5d,e). Thus, there is a strong correlation between the improved cell capacities and the value of *R*_CT_ of all cells, which (with the relative development of the external surface) seems to be a crucial parameter for CAG based anodes performance. Both CAG_PS and CAG_MS materials, after heat treatment at 700 °C, present optimal working parameters during galvanostatic charge-discharge tests, which is reflected in the lowest values of *R*_CT_ calculated by EIS. The highest *R*_CT_ values for all materials are achieved for those calcined at 900 °C. This can also be attributed to a high surface area (presented in N_2_-BET measurements) that hinders charge transfer reactions, probably through internal breakages occurring in too extensively developed aerogels.

## 4. Conclusions

In this work, the impact of pyrolysis temperature on the electrochemical, structural, and morphological properties of carbon aerogels derived from potato, maize, and rice starches has been investigated. The results show that there is a correlation between the surface evolution of starch based carbon nanostructures and their electrochemical behavior in Li-ion cell with regard to thermal treatment. It can be noted that the carbon porous structure and the textural parameters depend strongly on carbonization temperature; when it increases (up to 900 °C), the specific and external surface area, volume of micropores, and total pore volume also rise. Above 900 °C, all these mentioned values start to decline until the complete collapse of the pore structures at 1600 °C. This effect is also reflected in the electrochemical behavior of aerogels. It is likely that most of the pores of the samples heated at 600 °C were not completely exposed and accessible, therefore these materials exhibit worse electrochemical properties than carbons obtained at 700 °C. As the temperature rises, the violent release of volatile specious could be observed, and at the same time new pores may be formed. Even though the maximum BET surface areas was achieved at about 900 °C for all CAG materials, the highest relative development of the external surface was obtained for the samples pyrolysed at 700 °C. All these issues are in good agreement with the electrochemical measurements in which carbon aerogels obtained at 700 °C gained the highest specific capacities and the lowest charge transfer resistances. These effects are also in accordance with the “falling cards model”, which postulates that too high thermal treatment temperature leads to the replacement of small pores with larger ones due to the coalescence of the pores [34,35]. By further analyzing the behavior of materials prepared at higher temperatures (such as 1600 °C), it is seen that the carbon aerogels show worse performance in comparison to the rest of the samples. This is also connected with the widening of the pores, which results in the practical destruction and collapse of the pore structure and a sintering effect of the material grains [36].

To sum up, all presented results suggest that optimally developed structure, with the appropriate size and number of open pores, strongly depends on pyrolysis temperature. In the course of the research, the optimal pyrolysis temperature for starch based aerogel precursors was established at 700 °C. What is more, it can be noted that changes in the botanical origin of starch translate into the differences in the electrochemical behavior of the obtained carbon aerogels (the CAG_MS_700 electrode demonstrates much better electrochemical performance than the CAG_PS_700 electrode and a slightly better performance than the CAG_RS_700 electrodes in terms of higher specific capacities under various current loads). Therefore, we would like to indicate the fact that all mentioned parameters (such as material nature, its architecture, specific area, or the degree of order and disorder) are crucial for tailoring the electrochemical properties of carbon aerogels.

## Figures and Tables

**Table 1:** Textural parameters of the samples obtained by nitrogen sorption measurements.

**CAG_PS**	***S*** **_BET_** **[m^2^/g]**	***S*** **_EXT_** **[m^2^/g]**	***V*** **_MIC_** **[cm^3^/g]**	**V_TOT_** **(*p*/*p*_0_** **=** **0.99) [cm^3^/g]**	***S*** **_EXT_** **/*S*_BET_** **[-]**	***V*** **_MIC_** **/*V*_TOT_** **[-]**
600	371	19	0.179	0.213	0.05	0.83
700	302	73	0.116	0.187	0.24	0.62
900	569	93	0.187	0.258	0.16	0.72
1200	287	31	0.104	0.127	0.11	0.81
1600	29	30	0.001	0.098	-----	-----
**CAG_RS**	***S*** **_BET_** **[m^2^/g]**	***S*** **_EXT_** **[m^2^/g]**	***V*** **_MIC_** **[cm^3^/g]**	***V*** **_TOT_** **(*p*/*p*_0_** **=** **0.99) [cm^3^/g]**	***S*** **_EXT_** **/*S*_BET_** **[-]**	***V*** **_MIC_** **/*V*_TOT_** **[-]**
600	221	10	0.107	0.118	0.04	0.91
700	520	143	0.149	0.277	0.27	0.53
900	1743	475	0.503	0.929	0.27	0.54
1200	342	60	0.112	0.284	0.17	0.39
1600	51	46	0.002	0.147	-----	-----
**CAG_MS**	***S*** **_BET_** **[m^2^/g]**	***S*** **_EXT_** **[m^2^/g]**	***V*** **_MIC_** **[cm^3^/g]**	***V*** **_TOT_** **(*p*/*p*_0_** **=** **0.99) [cm^3^/g]**	**S_EXT_/S_BET_** **[-]**	***V*** **_MIC_** **/*V*_TOT_** **[-]**
600	169	6	0.083	0.092	0.03	0.90
700	549	167	0.152	0.310	0.30	0.49
900	657	136	0.205	0.327	0.21	0.63
1200	178	20	0.062	0.077	0.11	0.81
1600	31	29	0.001	0.079	-----	-----

Where: *S*_BET_—specific surface area, *S*_EXT_—external surface area, *V*_MIC_—micropore volume, *V*_TOT_—total pore volume.
